# Lightweight and precise cell classification based on holographic tomography-derived refractive index point cloud

**DOI:** 10.1117/1.JBO.30.9.096501

**Published:** 2025-09-02

**Authors:** Haoyuan Wang, Difeng Wu, Miao Zheng, Zuoshuai Zhang, Weina Zhang, Jianglei Di, Liyun Zhong

**Affiliations:** a Guangdong University of Technology, Institute of Advanced Photonics Technology, School of Information Engineering, Guangzhou, China; b Guangdong University of Technology, Ministry of Education of China, Key Laboratory of Photonic Technology for Integrated Sensing and Communication, Guangzhou, China; c Guangdong University of Technology, Guangdong Provincial Key Laboratory of Information Photonics Technology, Guangzhou, China

**Keywords:** holographic tomography, refractive index, point cloud, cell classification, deep learning

## Abstract

**Significance:**

Accurate cell classification is essential in disease diagnosis and drug screening. Three-dimensional (3D) voxel models derived from holographic tomography effectively capture the internal structural features of cells, enhancing classification accuracy. However, their high dimensionality leads to significant increases in data volume, computational complexity, processing time, and hardware costs, which limit their practical applicability.

**Aim:**

We aim to develop an efficient and accurate cell classification method using 3D refractive index (RI) point cloud data obtained from holographic tomography, focusing on reducing computational complexity without sacrificing classification performance.

**Approach:**

We transformed 3D RI voxel data into point cloud representations using segmented equilibrium sampling to substantially decrease data volume while retaining crucial structural features. A deep learning model, named RI-PointNet++, was then specifically designed for RI point cloud data to enhance feature extraction and enable precise cell classification.

**Results:**

In experiments classifying the viability of HeLa cells, the proposed method achieved a classification accuracy of 93.5%, significantly outperforming conventional two-dimensional models (87.0%). Furthermore, compared with traditional 3D voxel-based models, our method reduced computational complexity by over 99%, with floating-point operations of only 1.49 G, thus enabling efficient performance even on central processing unit (CPU) hardware.

**Conclusions:**

Our proposed method provides an innovative, lightweight solution for 3D cell classification, highlighting the considerable potential of point cloud–based approaches in biomedical research applications.

## 1 Introduction

Precise cell classification and identification can help uncover cellular functions and capture subtle phenotypic variations associated with diseases, thus playing a crucial role in early disease diagnosis, drug development, and personalized medicine.^1^^–^^3^ Achieving high-precision cell classification and identification hinges on high-quality cellular imaging data. Among conventional two-dimensional (2D) imaging techniques, fluorescence microscopy stands out for its ability to label specific cellular components, facilitating high-accuracy cell classification.^4^ However, fluorescence microscopy is hindered by issues such as photobleaching and phototoxicity, which restrict its applicability for long-term observation of live cells.^5^ In contrast, label-free imaging techniques, such as bright-field, dark-field, and phase-contrast microscopy, avoid the interference caused by dye labeling on cell states.^6^ Nevertheless, these methods provide only 2D, qualitative cellular representations, limiting the precision and accuracy of cell classification.

As an advanced label-free quantitative phase imaging technique, holographic tomography reconstructs the three-dimensional (3D) refractive index (RI) distribution of the sample by collecting phase information from multiple angles.^7^^,^^8^ In addition, as a critical physical parameter reflecting intracellular composition, RI enables more precise characterization of differences among various cell types. Consequently, 3D RI data obtained through holographic tomography demonstrate significant advantages in capturing cellular features and enhancing classification performance.^9^^,^^10^ A challenge and a hot point are how to effectively extract features from these 3D RI data to achieve high-precision cell classification. Traditional approaches rely on manually extracting geometric and physical characteristics of cells such as volume, surface area, and sphericity. These features are then input into statistical models or basic machine learning algorithms such as support vector machines or random forests for classification.^11^^–^^15^ These methods are relatively straightforward to implement, but they heavily rely on human expertise and struggle to fully exploit the high-dimensional nature of 3D data, resulting in poor consistency and efficiency when handling complex cell types. With the rapid development of deep learning, its application in processing holographic tomograms has expanded significantly.^16^^–^^20^ Combining deep learning with 3D RI data has emerged as an effective approach to improve classification accuracy and automation.^21^ For example, Kim et al.^22^ utilized a DenseNet architecture to process 3D RI imaging data, achieving a single-measurement accuracy of 82.5% in classifying 19 pathogenic bacterial species. Similarly, Ryu et al.^23^ employed 3D RI data combined with deep learning to classify bone marrow white blood cell subtypes with an accuracy exceeding 96%. These examples demonstrate that integrating 3D RI data with deep learning can significantly enhance cell classification performance. However, the voxel-based 3D data models used in these methods require substantial storage capacity and computational resources.^24^ Moreover, the high complexity of voxel data imposes stringent requirements on the analysis process, including heavy reliance on hardware resources during model training and high computational costs during inference. Hence, finding a way to maintain classification performance while effectively reducing data volume and computational complexity remains a critical challenge in the practical application of combining 3D RI data with deep learning.

To address the aforementioned challenges, point cloud representation has emerged as a prominent focus in 3D data processing.^25^ As an efficient form of 3D data representation, point cloud technology uses discrete sets of points to describe 3D structures, significantly reducing data volume while preserving spatial geometric and morphological information. In recent years, point clouds have demonstrated exceptional performance in various computer vision tasks, including large-scale 3D scene reconstruction, object recognition, and classification.^26^^–^^28^ Introducing point cloud technology into the processing of 3D RI data in the microscopic domain holds the potential to reduce computational complexity while maintaining accurate capture of cellular spatial features.^29^

Here, we propose an efficient cell classification method using 3D RI point clouds to address the high computational complexity associated with voxel-based RI data in classification tasks. First, the 3D RI voxel data are transformed into point cloud representations using segmented equilibrium sampling, which significantly reduces data volume while ensuring balanced distribution of critical features. Subsequently, we developed an optimized PointNet++ network, named RI-PointNet++ (RPNet++), specifically designed for feature extraction from cellular RI point clouds. RPNet++ enables the automated and efficient extraction of critical features from point cloud data. To evaluate the practical applicability of our method, experiments were conducted on HeLa cells with varying viability to assess the classification accuracy and computational efficiency of RPNet++. Compared with traditional voxel-based 3D approaches, our proposed method achieves high-precision classification tasks without requiring extensive high-performance computing resources, demonstrating exceptional computational efficiency and classification performance. This innovative solution highlights the potential of 3D RI point cloud data in advancing cell classification applications.

## 2 Experiments

The experiments consisted of two main parts. First, the 3D RI distribution of cells was obtained using a holographic tomography reconstruction algorithm, followed by preprocessing to convert the voxel data into corresponding RI point cloud representations. Subsequently, the RPNet++ network was constructed and trained, taking the RI point cloud data as input to predict cell categories.

### 2.1 Acquisition of 3D RI Voxel Data

**Fig. 1:**
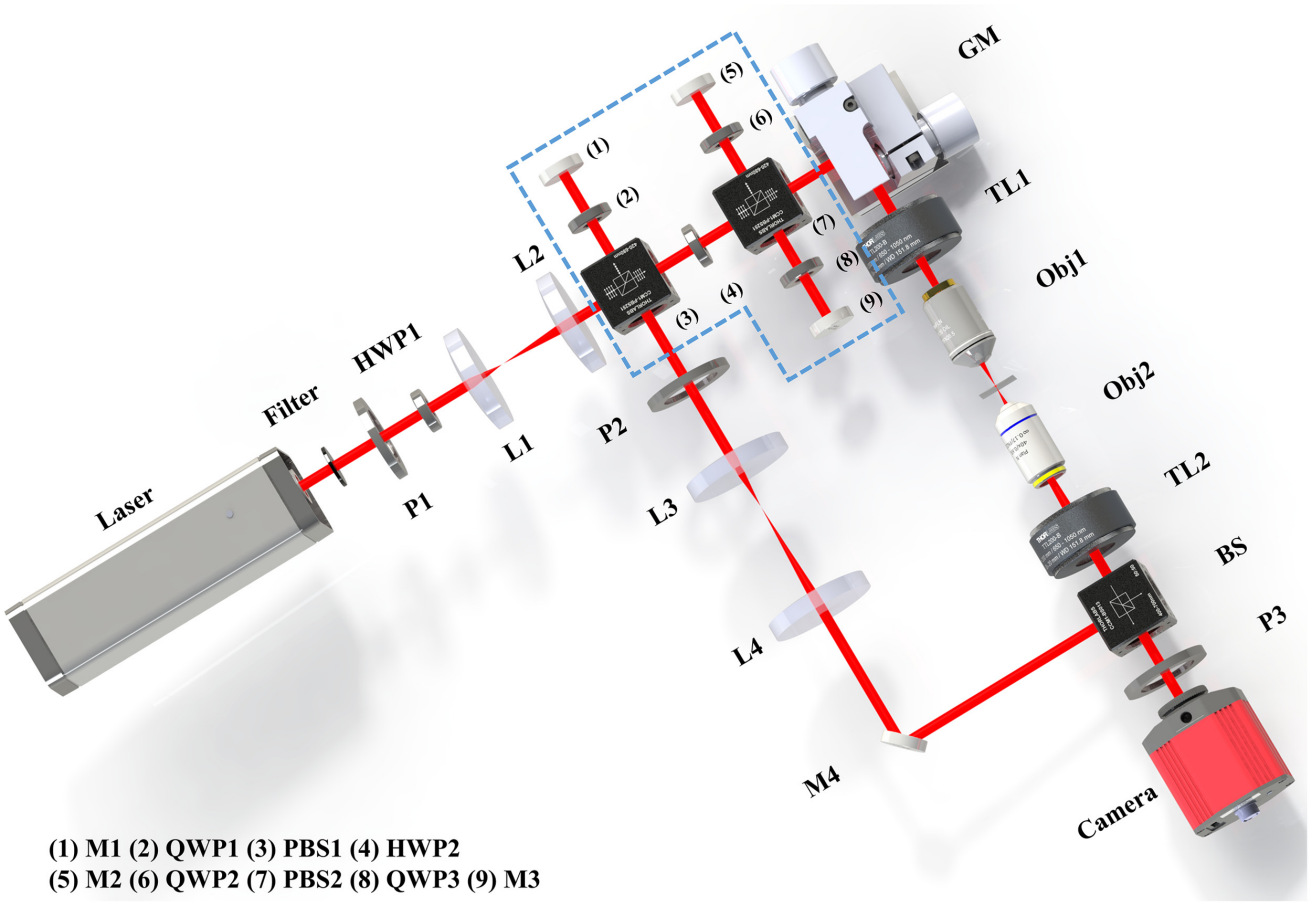
Schematic diagram of the holographic tomography optical setup. BS, beam splitter; GM, galvanometric mirrors; L, lens; M, mirror; Obj, microscope objective; P, polarizer; PBS, polarizing beam splitter; TL, tube lens; HWP, half-wave plate; QWP, quarter-wave plate.

The 3D RI voxel data of cells were acquired by capturing holograms from multiple angles, followed by reconstruction using a holographic tomography reconstruction algorithm. The experimental setup is shown in Fig. 1. The system is based on a Mach–Zehnder interferometric optical design and employs a low-coherence light source (central wavelength 641 nm, bandwidth 3 nm, and laser power 70 mW) as the illumination source. The laser beam is split into two parts by PBS1: one serves as the reference beam, whereas the other serves as the object beam to illuminate the sample. A 2D galvanometric mirror (GVS012, Thorlabs, Newton, New Jersey, United States) controls the object beam in a circular scanning pattern to illuminate the sample from 51 different angles (including one normal incidence), covering a numerical aperture of 0.65. The sample is positioned between a condenser objective lens (Obj1, PLN40X, Olympus, Tokyo, Japan) and an imaging objective lens (Obj2, UPLXAPO40XO, Olympus). The system uses a tube lens (TL2, f=180  mm) to project the image onto a monochromatic CMOS sensor (BFS-U3-16S2M-CS, 1440×1080  pixels, 3.45  μm/pixel). The total magnification of the system is 42.8×. The reference beam and object beam are combined at a non-polarizing beam splitter, with the optical path lengths precisely matched by adjusting mirrors M1 and M3 to meet interference conditions. The final magnified sample image with interference fringes is recorded by the CMOS sensor, capturing holograms at 51 angles within ∼5  s.

The acquired multi-angle holograms were processed using a holographic tomography reconstruction algorithm to reconstruct 3D holographic tomograms,^30^ yielding the 3D RI voxel data. To address the missing cone problem, non-negativity constraints were applied during reconstruction.^31^ The reconstructed data were processed using the optical diffraction tomography semi-automatic segmentation (ODT-SAS) algorithm,^32^ to isolate single-cell regions, with manual verification to ensure the accuracy of segmentation results. After segmentation, each single-cell dataset was cropped and standardized to a uniform voxel size of 320×320×210 to facilitate subsequent analysis.

### 2.2 Sample Preparation

HeLa cells (National Collection of Authenticated Cell Cultures, Shanghai, China) were thawed and maintained in Dulbecco’s Modified Eagle Medium (Gibco, Waltham, Massachusetts, United States) supplemented with 10% fetal bovine serum (Pricella) and 1% penicillin–streptomycin. Cultures were incubated at 37°C in a 5% ${\mathrm{CO}}_{2}$ humidified atmosphere, with medium changes every 2 to 3 days. Upon reaching ∼80% confluence, cells were trypsinized (Gibco), passaged two to three times, and then seeded onto fresh dishes for a further 24 h. Cell viability is defined as the capacity of cells to sustain essential physiological functions, including energy metabolism, proliferative potential, and specialized activities, under defined conditions, thereby reflecting their survival and functional integrity. As apoptosis progresses, cellular phosphatidylserine gradually translocates to the outer leaflet of the plasma membrane. When specifically labeled with Annexin V–FITC, the resulting fluorescence intensity increases progressively as cell viability declines. Furthermore, propidium iodide enables the staining and identification of necrotic cells. Therefore, by measuring the fluorescence intensity of labeled cells, we can classify their viability. In our experiments, HeLa cells were treated with 20  nmol/L paclitaxel to induce apoptosis. As paclitaxel exposure time increased, cell viability progressively declined. Untreated cells were defined as high-viability cells. After 3 h of treatment, fluorescence intensity rose markedly, and these cells were classified as moderate-viability cells. At 6 h, fluorescence intensity reached its peak, and the cells were defined as low-viability cells. Fluorescence intensity profiles for the three cell viability groups are available in Fig. S1 in the Supplementary Material. Finally, all groups were fixed with 4% paraformaldehyde for 20 min at 4°C and then washed twice each with phosphate-buffered saline (Tianjin Haoyang Biological Manufacture Co., Ltd., Tianjin, China) and ultrapure water (Milli-Q Millipore system) to remove residual fixative and impurities before holographic tomography imaging.

### 2.3 Acquisition of 3D RI Point Cloud Data

After reconstructing the 3D RI voxel data from the holographic tomography system, we sampled the data to generate 3D RI point cloud data for subsequent analysis. The specific procedure is as follows:

The 3D RI voxel data V are represented as

$$V=\{v_{i}=(x_{i},y_{i},z_{i},n_{i})|i=1,2,\ldots ,N\},$$ (1)

where $\left(x_{i},y_{i},z_{i}\right)$ are the spatial coordinates of the i’th voxel, $n_{i}$ is the corresponding RI value, N is the total number of voxels, and $v_{i}$ represents a single voxel data point. Through sampling, the point cloud data P are obtained

$$P=\{p_{j}=(x_{j},y_{j},z_{j},n_{j})|j=1,2,\ldots ,M\},$$ (2)

where $\left(x_{j},y_{j},z_{j}\right)$ are the spatial coordinates of the j’th point in the point cloud, $n_{j}$ is the corresponding RI value, M is the total number of points in the point cloud, and M<N. The point $p_{j}$ represents a single data point.

The sampling method is crucial for extracting meaningful features from 3D data. To comprehensively capture critical subcellular structures within cells, such as nucleoli and lipid droplets,^33^^,^^34^ while ensuring balanced representation of regions with different RI values, we proposed a segmented equilibrium sampling method. The method first normalizes the RI range and divides it into L intervals (in this study, L=6). An equal number of points are sampled from each interval to ensure balanced coverage across different RI regions. If a specific interval lacks sufficient points, additional points are randomly supplemented from other intervals to maintain overall balance.

**Fig. 2:**
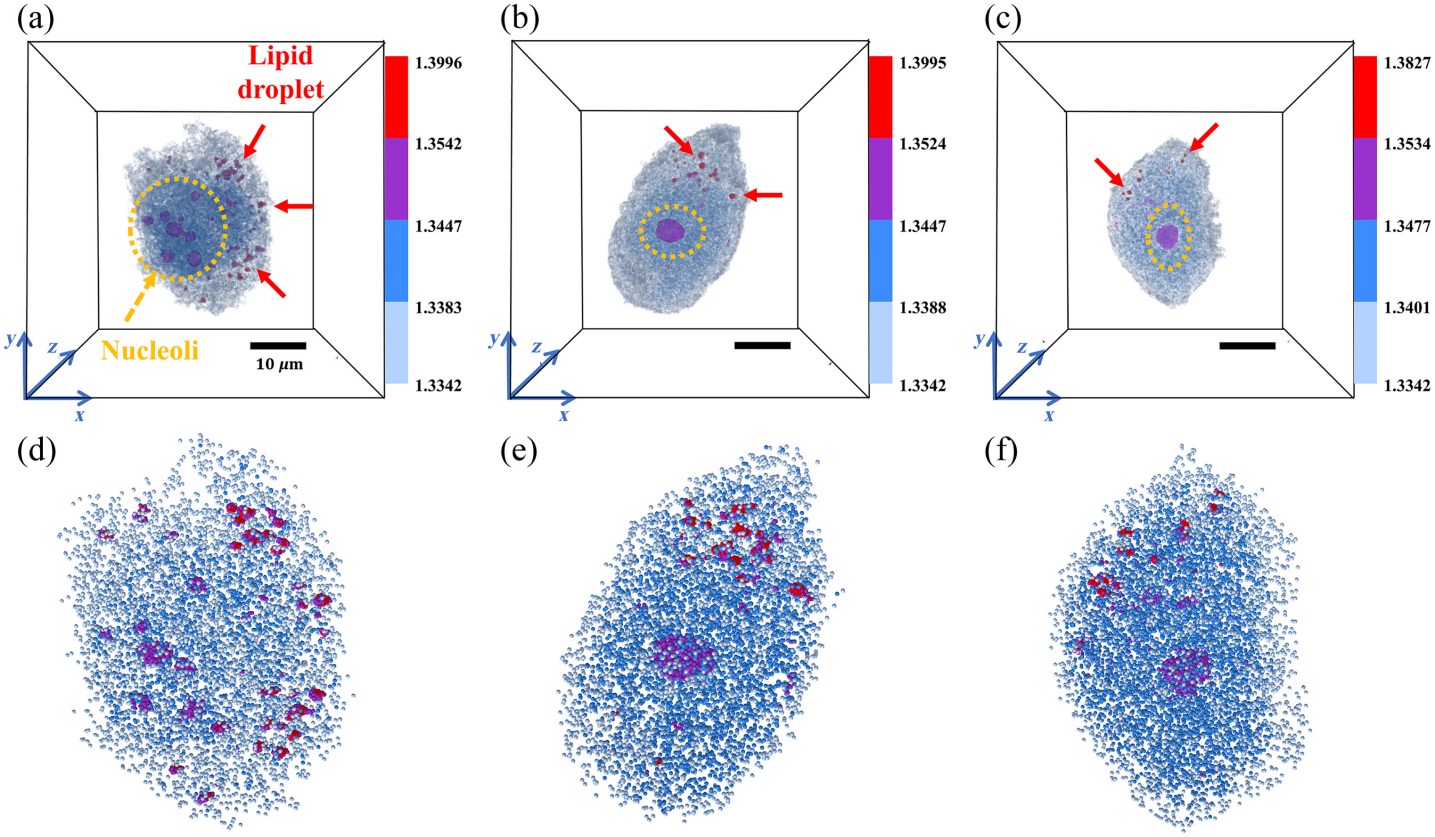
3D RI voxel data and corresponding point cloud representations for HeLa cells at different viability states. (a)–(c) 3D RI voxel visualizations of HeLa cells in (a) high viability, (b) moderate viability, and (c) low viability states. Yellow dashed circles indicate the nucleoli regions, and red arrows highlight the lipid droplets. Visualizations include xyz coordinate axes for spatial orientation, a 10-μm scale bar, and a color bar representing RI values. (d)–(f) Point cloud visualizations corresponding to panels (a)–(c). The point clouds are normalized to fit within a unit sphere and use the same spatial orientation and RI range as in panels (a)–(c).

The original 3D RI voxel data V have a total voxel count N ranging from $5.0\times 10^{5}$ to $4.0\times 10^{6}$. As shown in Figs. 2(a)–2(c), the original 3D RI voxel data of HeLa cells in different viability states have voxel counts of $1.86\times 10^{6}$, $2.43\times 10^{6}$, and $1.33\times 10^{6}$, respectively. The voxel data are massive in volume and computationally intensive, leading to high complexity in analysis. The yellow dashed lines indicate the nucleoli regions, whereas the red arrows mark the lipid droplets, both representing critical subcellular structures. Using the segmented equilibrium sampling method, the voxel data V were converted into point cloud data P with a uniform point count $M=1.0\times 10^{4}$. As shown in Figs. 2(d)–2(f), the sampled point cloud data significantly reduce data volume while still clearly reflecting the internal features of HeLa cells in different viability states, including the spatial distribution and RI differences of nucleoli and lipid droplets. The point cloud data obtained through equilibrium sampling maintain consistency with the spatial structure and key region features of the original voxel data, providing a more concise and effective input for subsequent classification models.

### 2.4 RPNet++ Network Architecture

**Fig. 3:**
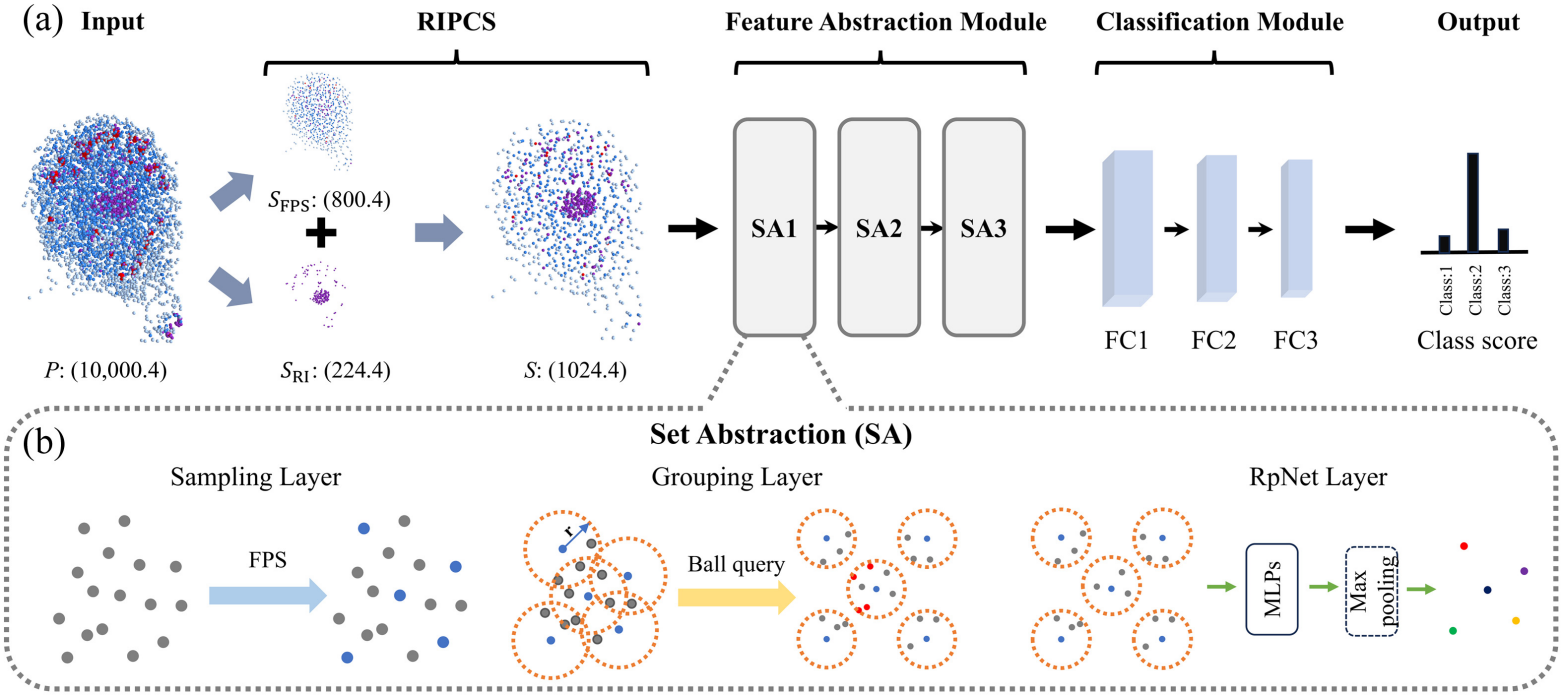
Schematic of the RPNet++ network architecture. (a) Overall framework of RPNet++, consisting of the RIPCS, feature abstraction module, and classification module. (b) SA: comprising sampling, grouping, and RPNet layers. FC, fully connected; FPS, farthest point sampling; MLP, multilayer perceptron; RIPCS, refractive index point cloud selector.

We propose a specialized network architecture called RPNet++, designed specifically for feature extraction and classification of 3D RI point cloud data of cells. RPNet++ consists of three main modules: the refractive index point cloud selector (RIPCS), the feature abstraction module, and the classification module. As shown in Fig. 3(a), the input point cloud data first undergo sampling and processing through RIPCS. Subsequently, the feature abstraction module extracts deep-level features, and finally, the classification module outputs the predicted cell categories.

#### 2.4.1 Refractive index point cloud selector

In point cloud classification networks, the core function of the RIPCS module is to extract key points from the original point cloud data P to form a training point set S. Unlike in the earlier stage, where segmented equilibrium sampling was used to convert 3D voxel data into lightweight point clouds, RIPCS further refines and selects the point cloud data. It standardizes the point cloud to a fixed number of K, thereby reducing computational complexity and memory requirements and accelerating the training and inference processes. In this paper, the number of sampled points in RIPCS was set to K=1024, following standard practice in many point cloud classification networks such as PointNet++^35^ and Point transformer^36^ to ensure compatibility with widely used model architectures. The classification performance metrics for varying point counts K are shown in Table S1 in the Supplementary Material. In addition, RIPCS introduces data augmentation through randomized sampling points, enabling the model to encounter more diverse point cloud subsets during training iterations, significantly improving its generalization capability.

Specifically, the RIPCS module combines farthest point sampling (FPS) and refractive index interval–enhanced sampling (RIIES) to address issues of global coverage and key feature representation in point cloud sampling. First, FPS is used to select 800 points from the original point cloud P, generating a subset $S_{\mathrm{FPS}}$ with global spatial coverage to ensure the comprehensive representation of overall geometric features. The FPS method iteratively selects the farthest points in the point cloud,^35^ effectively capturing global geometric characteristics and ensuring uniform spatial distribution of sampling points, providing robust support for overall structural representation. However, as FPS lacks specificity in selecting particular regions, using it alone may lead to information loss. To complement FPS, the proposed RIIES technique focuses on taking values at a specific RI interval corresponding to a specific cell structure. In our work, the substances located in the specific RI interval (1.345 to 1.355) chosen by RIIES are known to correspond to nucleoli,^33^ a subcellular structure whose RI changes are strongly correlated with HeLa cell viability. It selects 224 points from the selected critical RI interval to generate the subset $S_{\mathrm{RI}}$, enhancing the coverage of the nucleolar regions within cells. If fewer than 224 points exist in the defined interval, existing points were replicated from within that region to meet the required number, ensuring consistency across samples. This enhanced sampling method effectively captures characteristic features closely related to the classification task, ensuring sufficient representation of information in critical RI intervals. By combining FPS and RIIES, a hybrid training point set $S=S_{\mathrm{FPS}}\cup S_{\mathrm{RI}}$ is generated. This approach preserves the global geometric features of the point cloud while emphasizing the regional characteristics of critical subcellular structures. It provides more comprehensive and reliable data support for targeted classification of specific cell categories in various classification tasks.

#### 2.4.2 Feature abstraction module

The feature abstraction module is designed to progressively extract multi-level features from point cloud data. As shown in Fig. 3(a), the module consists of three set abstraction (SA) levels, with each level comprising a sampling layer, a grouping layer, and an RPNet layer [Fig. 3(b)]. Through this hierarchical feature extraction approach, the module captures both local and global key information step by step. In the sampling layer, the FPS method is used to select $n_{\text{center}}$ center points from the input point set, ensuring their uniform spatial distribution. This reduces information loss caused by uneven point cloud distributions. Following this, the grouping layer uses the selected center points as cores and applies the ball query method to find all neighboring points within a specified radius r, forming local region point sets. The radius r is defined unitless in the normalized coordinate space, where all input point clouds are scaled to fit within a unit sphere to ensure consistent local region definitions across samples regardless of their original physical size. To limit computational complexity and ensure consistency across local regions, the number of points in each region is capped at $n_{\text{sample}}$. Each local region point set contains the spatial coordinates and RI values of the points. The RPNet layer performs hierarchical feature extraction on the local region point sets. First, the coordinates of each point are transformed into local coordinates relative to the center point, eliminating the influence of global positioning and retaining only local geometric relationships. Next, multiple multilayer perceptrons (MLPs) are used to perform pointwise mapping on the local coordinates and RI values, generating high-dimensional feature representations. These pointwise features within each set abstraction module are then aggregated for each local region using max pooling, resulting in compact global feature vectors that comprehensively describe the geometric structure and RI information of the region.

The specific parameters of the feature abstraction module are shown in Table 1. By progressively abstracting features, the module effectively extracts both local and global characteristics, addressing the complexity of cellular RI point clouds and significantly improving classification accuracy and model robustness.

**Table 1:** Specific parameters of the feature abstraction module.

Level	Layer	Parameters
Set abstraction 1	Sampling 1	$n_{\mathrm{center}}=512$
	Grouping 1	r=0.15, $n_{\text{sample}}=32$
	RPNet 1	MLPs: [64, 64, 128]
Set abstraction 2	Sampling 2	$n_{\mathrm{center}}=256$
	Grouping 2	r=0.3, $n_{\mathrm{sample}}=64$
	RPNet 2	MLPs: [128, 128, 256]
Set abstraction 3	Grouping 3	Group all points
	RPNet 3	MLPs: [256, 512, 1024]

#### 2.4.3 Classification module

The classification module is designed based on the standard classification layer of PointNet++,^35^ consisting of three fully connected layers. This module maps the global feature vector extracted by the feature abstraction module to the final class probability distribution. In the classification workflow, the input global feature vector is progressively dimensionally compressed through the fully connected layers. Batch normalization is applied to enhance model stability and training efficiency, whereas the ReLU activation function is used to improve feature expressiveness. Dropout is employed to suppress overfitting. Finally, the features are mapped to the number of target classes and converted into a logarithmic probability distribution using the log-softmax activation function. The classification result is determined by the class with the highest probability. The specific parameters of the classification module are shown in Table 2.

**Table 2:** Specific parameters of the classification module.

Layer	Input dimension	Output dimension	Dropout rate	Activation
Fully connected 1	1024	512	0.3	ReLU
Fully connected 2	512	256	0.4	ReLU
Fully connected 3	256	3	—	log-softmax

### 2.5 RPNet++ Training and Dataset Preparation

The training of RPNet++ utilizes the AdamW optimizer,^37^ with an initial learning rate of 0.001 and a weight decay factor of 0.0001, for a total of 200 training epochs. The batch size is set to 16 to balance computational efficiency and model performance. The learning rate schedule employs the cosine annealing method,^38^ dynamically reducing the learning rate using a cosine function to ensure stable model convergence. The model is optimized using the cross-entropy loss function with label smoothing (α=0.1). To enhance the generalization ability of the model, various data augmentation strategies are applied during training with a 50% probability. These strategies include random rotation, random perturbation, and the addition of Gaussian noise.^39^ All the input cell point cloud data are normalized to unit spheres before being fed into the network, which allows for consistent spatial scaling across cells of different physical sizes, thereby making the selection of a fixed number of K transferable across different datasets. The dataset consists of 456 HeLa samples in three viability states: 123 samples with high viability, 179 samples with moderate viability, and 154 samples with low viability. The dataset is split into a training set and a test set at a ratio of 8:2, with 364 samples in the training set and 92 samples in the test set. During the model tuning phase, k-fold cross-validation is used to validate the robustness and generalization ability of the configuration, ensuring model stability across different data splits. The final classification performance evaluation is conducted on a single data split with fixed configurations after model tuning, using a consistent random seed to guarantee result reproducibility. All experiments are conducted on a workstation equipped with an Intel Core i9-13900K processor and an NVIDIA GeForce RTX 4090 graphics processing unit (GPU). The neural network implementation is based on the PyTorch framework.

## 3 Results and Discussion

In this section, we discuss the results in the following three aspects. In Sec. 3.1, we validate the effectiveness of segmented equilibrium sampling in optimizing point cloud representation. In Sec. 3.2, we analyze the impact of point cloud sampling and RPNet++ configuration on classification performance. In Sec. 3.3, we compare the classification performance and computational efficiency of RPNet++ with traditional 2D and 3D models.

For the evaluation of classification performance, the following metrics are used: accuracy, recall, precision, F1-score, and area under the receiver operating characteristic curve (AUC). Accuracy measures the proportion of correctly predicted samples, reflecting the overall correctness of the model. Recall indicates the model’s ability to identify positive samples, focusing on sensitivity to true positives. Precision evaluates the proportion of correctly predicted positive samples among all predicted positives, emphasizing the reliability of predictions. The F1-score balances recall and precision, making it particularly suitable for tasks with imbalanced class distributions. AUC, derived from the receiver operating characteristic (ROC) curve, provides a comprehensive assessment of the model’s classification capability, reflecting its stability and reliability across different thresholds. In addition, to evaluate the complexity and computational efficiency of the model, two key indicators are analyzed: parameters and floating-point operations (FLOPs). Parameters measure the size of the model, directly affecting storage requirements. FLOPs represent the computational workload during inference, indicating the computational complexity of the model. By comprehensively considering classification performance and computational complexity, the proposed method achieves a favorable balance between performance and efficiency.

### 3.1 Impact of Sampling Methods on Data Feature Representation

**Fig. 4:**
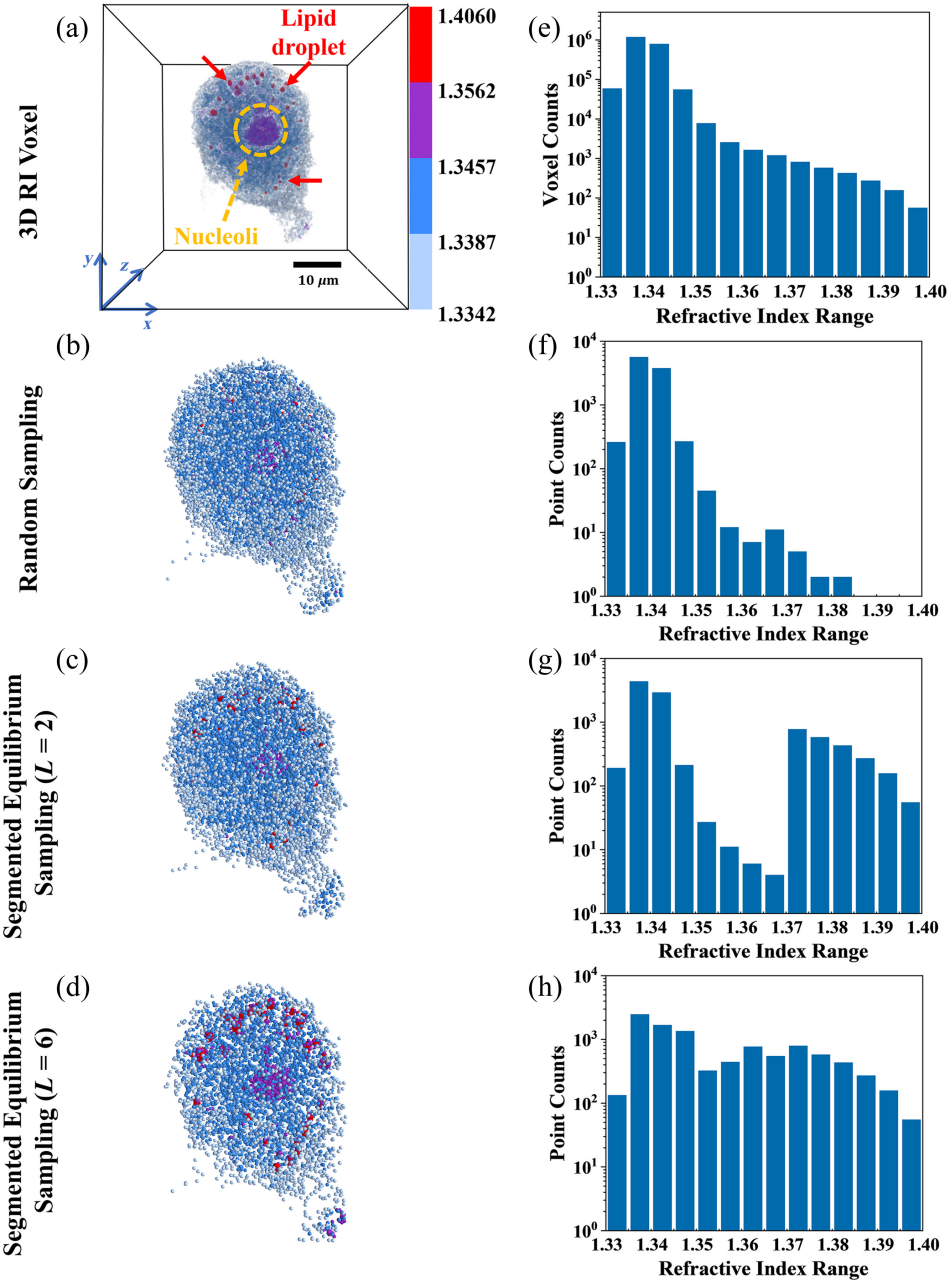
Comparison of 3D RI voxel data and point cloud representations under different sampling methods. (a) Visualization of the original 3D RI voxel data for HeLa cells. Yellow dashed circles indicate the nucleoli regions, and red arrows highlight the lipid droplets. The visualization includes xyz coordinate axes, a 10-μm scale bar, and a color bar for the RI range. (b)–(d) Point cloud visualizations generated using different sampling methods: (b) random sampling, (c) segmented equilibrium sampling with L=2, and (d) segmented equilibrium sampling with L=6. Point clouds are normalized to fit within a unit sphere, and spatial orientation and RI range are consistent with panel (a). (e) Logarithmic distribution of voxel counts across different RI intervals in the original voxel data. (f)–(h) Logarithmic distribution of point counts across different RI intervals for point clouds generated using (f) random sampling, (g) segmented equilibrium sampling with L=2, and (h) segmented equilibrium sampling with L=6.

To evaluate the effectiveness of the segmented equilibrium sampling method in processing RI point cloud data of cells, we compared it with the traditional random sampling method and examined how different values of L affect the feature representation of point cloud data. Figure 4 presents the 3D RI voxel data of HeLa cells and the point clouds generated by different sampling methods, along with the visualization and RI distributions for both the voxel data and the point clouds.

From the visualization results of 3D data [Figs. 4(a)–4(d)], the 3D RI voxel data of HeLa cells [Fig. 4(a)] reveal the complex spatial distribution of refractive indices within the cell, where the yellow circles and red arrows highlight the nucleoli and lipid droplet regions, respectively. Point clouds generated by random sampling [Fig. 4(b)] show excessive sampling of low-RI regions and insufficient sampling of high-RI regions, resulting in inadequate representation of critical structures such as lipid droplets and nucleoli. In contrast, the segmented equilibrium sampling method [Figs. 4(c) and 4(d)] significantly improves the distribution of the point cloud by reasonably dividing RI intervals and uniformly sampling within each interval. When L=2 [Fig. 4(c)], the point cloud achieves preliminary coverage of major features across different RI intervals. As L=6 [Fig. 4(d)], the point cloud achieves more balanced coverage across intervals, clearly exhibiting details of substructures such as lipid droplets and nucleoli, thereby enhancing feature representation capabilities.

From the comparison of RI distributions across intervals [Figs. 4(e)–4(h)], the 3D voxel data of HeLa cells [Fig. 4(e)] show an imbalance in RI distribution, with the voxel count in the low-RI interval (1.33 to 1.345) exceeding other intervals by three to four orders of magnitude. This imbalance directly affects the point cloud generated by random sampling [Fig. 4(f)], where the proportion of points in the low-RI regions is excessively high, and other intervals are severely underrepresented. After applying segmented equilibrium sampling [Figs. 4(g) and 4(h)], as L increases, the point cloud coverage across RI intervals becomes progressively more balanced. By L=6 [Fig. 4(h)], the point cloud achieves a more reasonable distribution across intervals, significantly increasing the proportion of points in high-RI intervals. This comprehensive coverage of the cell’s internal features enhances the overall expressiveness of the point cloud in capturing the cell’s complex structures.

Segmented equilibrium sampling effectively addresses the issue of over-sampling in low-RI regions associated with random sampling, significantly improving the ability of point cloud data to represent the complex internal structures of cells. This method optimizes the balance of point cloud distributions, providing more representative input data for subsequent classification experiments. The specific impact on classification performance and the selection of the L parameter will be elaborated in Sec. 3.2.

### 3.2 Impact of Point Cloud Sampling and RPNet++ Configuration on Classification Performance

To comprehensively evaluate the impact of point cloud sampling methods and RPNet++ module configurations on classification performance, we designed eight experiments focusing on three key aspects: input dimensions, voxel-to-point cloud sampling methods, and point cloud selection strategies in RIPCS. The analyses are detailed as follows:

1. Input dimensions: We compared the classification performance of two input configurations: one including only 3D spatial coordinates (x,y,z) and the other including both 3D spatial coordinates and corresponding RI information (x,y,z,n).
2. Voxel-to-point cloud sampling methods: We analyzed the differences in classification performance between random sampling and segmented equilibrium sampling. Furthermore, we investigated how different L values in segmented equilibrium sampling impact performance.
3. Point cloud selection strategies in RIPCS: We evaluated the performance differences using FPS only, RIIES only and employing a hybrid sampling strategy that combines FPS with RIIES. The analysis focused on the optimization effects of these methods on the feature representation capabilities of point clouds.

**Fig. 5:**
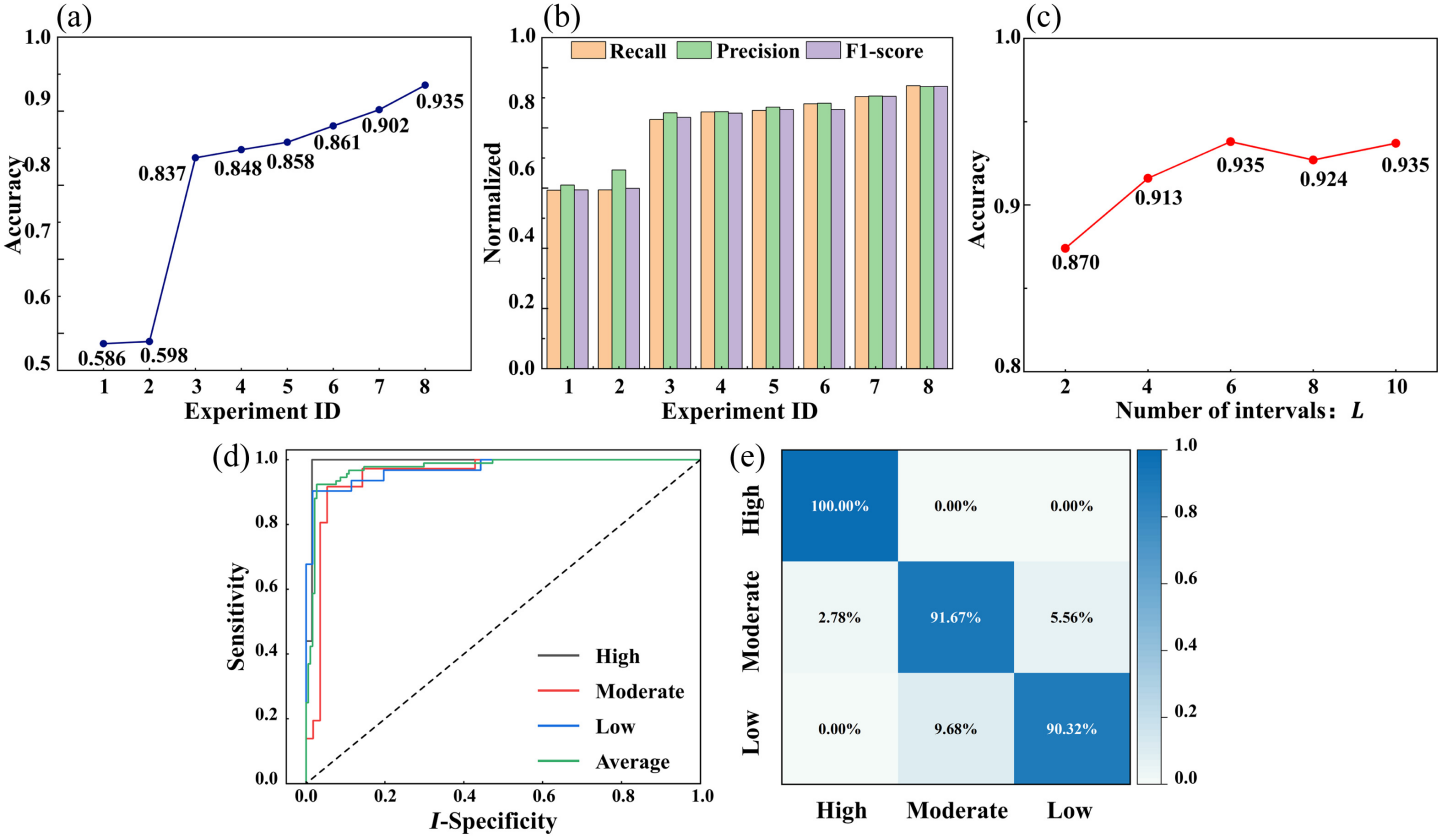
Experimental results and classification performance. (a) Comparison of classification accuracy across eight experiments. (b) Comparison of recall, precision, and F1-score for eight experiments. (c) Impact of different values of L on classification accuracy. (d) ROC curves and average AUC for the proposed method (4D-S-Hybrid), with an average AUC of 0.9745. (e) Confusion matrix for the proposed method (4D-S-Hybrid).

Details of the experimental settings are presented in Table 3, and the results under different configurations are illustrated in Fig. 5.

**Table 3:** Experimental settings for the classification experiments.

Experiment ID	Method name	Input dimension	Voxel-to-point cloud sampling method	RIPCS strategy
1	3D-R-FPS	x, y, z	Random sampling	FPS-only sampling
2	3D-S-FPS	x, y, z	Segmented equilibrium sampling	FPS-only sampling
3	4D-R-FPS	x, y, z, n	Random sampling	FPS-only sampling
4	4D-S-FPS	x, y, z, n	Segmented equilibrium sampling	FPS-only sampling
5	4D-R-RIIES	x, y, z, n	Random sampling	RIIES-only sampling
6	4D-S-RIIES	x, y, z, n	Segmented equilibrium sampling	RIIES-only sampling
7	4D-R-Hybrid	x, y, z, n	Random sampling	Hybrid sampling
**8**	**4D-S-Hybrid**	x, y, z, n	**Segmented equilibrium sampling**	**Hybrid sampling**

Note: Bold represent our proposed method.

#### 3.2.1 Impact of input dimensions on classification performance

The dimensionality of input information determines the types of features that can be extracted from point cloud data and the representational capacity of the classification model. When only 3D spatial coordinates (x,y,z) are provided, the point cloud data can only convey geometric distributions, relying on spatial relationships among points for feature extraction. In this scenario, the network struggles to effectively capture the complex physical state differences within cells. By incorporating RI information, the point cloud data gain the ability to describe physical properties associated with cellular components. Local features can reflect variations in RI distributions within specific regions of the point cloud, such as microscopic changes in the nucleoli and lipid droplet regions, whereas global features capture the overall trends in cellular RI changes, aiding the model in distinguishing cells with different viability states.

The experimental results demonstrate that the inclusion of RI information significantly enhances classification performance. Under random sampling conditions, the classification accuracy improved from 58.6% to 83.7% [Fig. 5(a), 3D-R-FPS versus 4D-R-FPS]. Similarly, under segmented equilibrium sampling conditions, the accuracy increased from 59.8% to 84.8% [Fig. 5(a), 3D-S-FPS versus 4D-S-FPS]. These results indicate that RI information not only enriches the expressive power of point cloud features but also significantly enhances the representation of internal cellular structures and states, thereby improving classification performance.

#### 3.2.2 Impact of voxel-to-point cloud sampling methods on classification performance

The voxel-to-point cloud sampling method directly determines the quality of the original point cloud data, thereby influencing classification performance. Random sampling does not account for the balance of RI distributions, leading to insufficient representation of high-RI regions and limiting the classification model’s ability to accurately describe internal cellular structures. In contrast, segmented equilibrium sampling ensures a more comprehensive coverage of the cell’s complex structural features by evenly distributing points across different RI intervals.

Experimental results demonstrated that segmented equilibrium sampling significantly improved the representation quality of point clouds. Under the FPS strategy alone, segmented equilibrium sampling increased classification accuracy from 83.7% to 84.8% and the F1-score from 0.835 to 0.849 [Figs. 5(a) and 5(b); 4D-R-FPS versus 4D-S-FPS]. However, as the FPS strategy primarily focused on global spatial coverage, it failed to sufficiently capture details in key feature regions, resulting in relatively limited performance improvements. The RIIES strategy was used to seize the detailed changes in key areas to enhance perception ability. Under the RIIES strategy alone, segmented equilibrium sampling increased classification accuracy from 85.8% to 88.0% and the F1-score from 0.861 to 0.881 [Figs. 5(a) and 5(b); 4D-R-RIIES versus 4D-S-RIIES]. To further enhance classification performance, segmented equilibrium sampling was combined with a hybrid sampling strategy, which improves the capture of critical feature regions and global information in the meantime. This combination achieved substantial performance gains, with classification accuracy increasing from 90.2% to 93.5% and the F1-score improving from 0.905 to 0.938 [Figs. 5(a) and 5(b); 4D-R-Hybrid versus 4D-S-Hybrid]. These improvements demonstrate that merely optimizing the sampling method is insufficient to fully exploit the potential of point cloud features. Instead, combining global coverage and enhanced sampling in critical regions significantly improves the representation of key areas in the point cloud, thereby boosting classification performance.

In addition, to validate how different L values in segmented equilibrium sampling influence classification performance, we analyzed the sampling results shown in Fig. 5(c). Initially, as L increased, the model’s classification accuracy improved, demonstrating the effectiveness of the segmented equilibrium sampling method. The model achieved optimal performance across all metrics (accuracy of 93.5%) when L=6, with balanced point cloud coverage across RI intervals and sufficient representation of high-RI regions such as lipid droplets and nucleoli. However, further increasing L provided diminishing returns, as excessively fine divisions offered limited marginal gains. To balance point cloud distribution and computational efficiency, this study selected L=6 as the final parameter for RI interval division.

#### 3.2.3 Impact of RIPCS strategy on classification performance

The size, number, and RI variations of the nucleoli are critical features for classifying cell viability.^33^^,^^40^ The RIPCS enhances the expression of key features in point cloud data by incorporating RIIES, which specifically captures RI information in nucleolar regions. Compared with relying solely on the global geometric coverage provided by FPS and on the details in key feature regions by RIIES, the hybrid sampling strategy combines the advantages of global coverage with localized enhancement, offering a more comprehensive representation of critical features within the point cloud.

Experimental results demonstrate that the hybrid sampling strategy significantly improves classification performance. Under random sampling conditions, classification accuracy increased from 83.7% by FPS only or 85.8% by RIIES only to 90.2%, and the F1-score improved from 0.835 by FPS only or 0.861 by RIIES only to 0.905 [Figs. 5(a) and 5(b); 4D-R-FPS and 4D-R-RIIES versus 4D-R-Hybrid]. When combined with segmented equilibrium sampling, hybrid sampling further enhanced classification accuracy to 93.5% and the F1-score to 0.938 [Figs. 5(a) and 5(b); 4D-S-FPS and 4D-S-RIIES versus 4D-S-Hybrid]. It is worth noting that the RIPCS module is a random process, which involves stochasticity in both the FPS and the RIIES processes. To evaluate the effect of this randomness, 10 independent experiments were performed with different RIPCS sampling realizations, whose results showed a mean accuracy of 92.97% with a standard deviation of 0.71%, as shown in Fig. S2 in the Supplementary Material, indicating that the RICPS model is robust to the randomness in point selection. These findings indicate that the hybrid sampling strategy not only retains the advantages of FPS in achieving global spatial coverage but also strengthens the representation of high-RI regions, such as the nucleoli. This dual capability enables the model to better capture critical cellular features, resulting in substantial improvements in classification performance.

#### 3.2.4 Performance summary of the optimal configuration

The experimental results of the proposed final method (4D-S-Hybrid) are shown in Figs. 5(d) and 5(e), further demonstrating its excellent classification performance. Figure 5(d) illustrates the ROC curves and their corresponding AUC values. The average AUC reached 0.9745, with AUC values of 0.9916, 0.9514, and 0.9720 for high viability, moderate viability, and low viability, respectively. The confusion matrix shown in Fig. 5(e) highlights the classification accuracy for each viability state. High viability cells achieved a classification accuracy of 100%, whereas moderate viability and low viability cells attained accuracies of 91.67% and 90.32%, respectively. The proposed method leverages the synergistic effect of RI information and optimized sampling strategies, significantly enhancing the ability of point cloud data to represent the complex features of cells. Furthermore, it demonstrates notable advantages in classification accuracy and model robustness, providing a reliable and innovative solution for efficient cell classification tasks.

### 3.3 Comparison of RPNet++ with 2D and 3D Methods

To comprehensively evaluate the performance of RPNet++ in processing 3D point cloud data, four representative deep learning models were selected for comparison, including three 2D models and one 3D model. DenseNet121-2D^41^ is well-suited for classification tasks requiring high accuracy due to its parameter-efficient design. ResNet101-2D^42^ demonstrates excellent feature extraction capabilities with its deep network structure, making it ideal for handling complex tasks. MobileNet-2D^43^ features a lightweight design optimized for efficient operation in resource-constrained environments. In addition, DenseNet121-3D is widely applied in holographic tomography classification tasks,^22^^,^^44^^,^^45^ delivering exceptional performance but with high computational resource demands.

For input data, the 2D models utilized three common input formats: maximum intensity projection (MIP) images, central slice images, and phase images. MIP images project the overall intensity distribution of the cell, capturing global features. Central slice images extract the core section of the 3D data, emphasizing local features. Phase images are a conventional input format commonly used in traditional microscopy. The 3D model directly employs a complete 3D holographic tomogram as input, capturing both global and internal structural complexity of the cell, thereby offering higher expressive power. These models and input designs provide a baseline for evaluating the classification performance of RPNet++.

The experimental results, summarized in Table 4, show that different input formats highlight distinct cellular features, directly influencing classification accuracy. The classification accuracies of DenseNet121-2D on central slice images, MIP images, and phase images were 85.9%, 84.8%, and 82.6%, respectively. ResNet101-2D achieved accuracies of 87.0%, 80.4%, and 80.4%, whereas MobileNet-2D achieved 80.4%, 78.3%, and 76.1%. However, the 3D model DenseNet121-3D, despite its potential, was constrained by computational resource limitations, achieving a classification accuracy of 82.6% in this experiment. This limitation is likely due to the small batch size (only two) caused by insufficient GPU memory during training, which adversely impacted parameter optimization and classification performance. In addition, large-scale 3D data processing has a strong hardware dependency, increasing experimental complexity.

**Table 4:** Classification results for RPNet++ and traditional 2D/3D models.

Model	Input data size	Parameters (M)	FLOPs (G)	Accuracy (%)
DenseNet121-2D	320 × 320	6.95	88.72	85.9
ResNet101-2D	320 × 320	42.50	250.88	87.0
MobileNet-2D	320 × 320	3.22	4.60	80.4
Densenet121-3D	320 × 320 × 210	11.25	882.56	82.6
**RPNet++**	**1024 × 4**	**1.47**	**1.49**	**93.5**
**RPNet++(CPU)**	**1024 × 4**	**1.47**	**1.49**	**92.4**

Note: Bold represent our proposed method.

In terms of parameters and FLOPs, RPNet++ demonstrated exceptional efficiency compared with traditional 2D and 3D models. The parameter count of DenseNet121-3D was 11.2 M, with FLOPs reaching 882.56 G, requiring substantial computational resources and reliance on high-performance GPUs during training. In contrast, RPNet++ had a parameter count of only 1.47 M, which is 13% of DenseNet121-3D, and FLOPs of just 1.49 G, over 99% lower than DenseNet121-3D. In addition, to ensure a fair comparison, we also assessed the computational overhead of the RIPCS module. The FLOPs of RIPCS were 0.1 G and did not significantly impact the efficiency of inference. Furthermore, RPNet++ not only operates efficiently on GPUs but also supports training and inference on central processing unit (CPU). When running on a CPU, RPNet++ achieved a classification accuracy of 92.4%, almost matching its GPU performance of 93.5%, demonstrating exceptional hardware adaptability. This capability significantly reduces hardware resource dependency and broadens its potential applications in a resource-constrained environment.

## 4 Conclusion

In summary, we combined holographic tomography and point cloud technology to propose an efficient three-dimensional cell classification method. Through an innovative voxel-to-point cloud conversion approach and a deep learning framework designed for point cloud feature extraction, our method provides a pathway for 3D cellular data analysis. During the data conversion phase, segmented equilibrium sampling effectively improved the balance of point cloud coverage across different RI intervals, ensuring that critical substructures such as nucleoli and lipid droplets in high-RI regions were adequately represented. Combined with feature enhancement strategies, the optimized point cloud data significantly improved the description of complex cellular structures. Experimental results demonstrated that the proposed method achieved a classification accuracy of 93.5% in the HeLa cell viability classification task, significantly outperforming traditional models while substantially reducing computational complexity. This method exhibits broad application potential in lightweight design and sparse data feature representation, offering efficient and reliable technical support for other cell classification tasks, functional state assessments, and related biomedical research.

## Supplementary Material

10.1117/1.JBO.30.9.096501.s01

## Data Availability

The data and code developed in this study are available upon reasonable request to the corresponding author.

## References

[1] Moen E. et al., “Deep learning for cellular image analysis,” Nat. Methods 16(12), 1233–1246 (2019).1548-7091 10.1038/s41592-019-0403-1 31133758 PMC8759575

[r2] Zhang X. et al., “Rapid and accurate identification of stem cell differentiation stages via SERS and convolutional neural networks,” Biomed. Opt. Express 15(5), 2753 (2024). BOEICL 2156-7085 10.1364/BOE.519093 38855654 PMC11161375

[3] Shu X. et al., “Artificial-intelligence-enabled reagent-free imaging hematology analyzer,” Adv. Intell. Syst. 3(8), 2000277 (2021). 10.1002/aisy.202000277

[4] Lichtman J. W. Conchello J.-A., “Fluorescence microscopy,” Nat. Methods 2(12), 910–919 (2005).1548-7091 10.1038/nmeth817 16299476

[5] Shaked N. T. et al., “Label-free biomedical optical imaging,” Nat. Photonics 17(12), 1031–1041 (2023). NPAHBY 1749-4885 10.1038/s41566-023-01299-6 38523771 PMC10956740

[6] Ghosh B. Agarwal K., “Viewing life without labels under optical microscopes,” Commun. Biol. 6(1), 559 (2023). 10.1038/s42003-023-04934-8 37231084 PMC10212946

[7] Wolf E., “Three-dimensional structure determination of semi-transparent objects from holographic data,” Opt. Commun. 1(4), 153–156 (1969). OPCOB8 0030-4018 10.1016/0030-4018(69)90052-2

[8] Jin D. et al., “Tomographic phase microscopy: principles and applications in bioimaging [Invited],” J. Opt. Soc. Amer. B 34(5), B64 (2017). JOBPDE 0740-3224 10.1364/JOSAB.34.000B64 29386746 PMC5788179

[9] Kim G. et al., “Holotomography,” Nat. Rev. Methods Primers 4(1), 51 (2024). 10.1038/s43586-024-00327-1

[10] Wang L. Li M. Hwang T. H., “The 3D revolution in cancer discovery,” Cancer Discov. 14(4), 625–629 (2024). 10.1158/2159-8290.CD-23-1499 38571426

[11] Pirone D. et al., “Identification of drug-resistant cancer cells in flow cytometry combining 3D holographic tomography with machine learning,” Sens. Actuators B: Chem. 375, 132963 (2023). 10.1016/j.snb.2022.132963

[r12] Pirone D. et al., “Label-free liquid biopsy through the identification of tumor cells by machine learning-powered tomographic phase imaging flow cytometry,” Sci. Rep. 13(1), 6042 (2023). SRCEC3 2045-2322 10.1038/s41598-023-32110-9 37055398 PMC10101968

[r13] Park S. et al., “Label-free tomographic imaging of lipid droplets in foam cells for machine-learning-assisted therapeutic evaluation of targeted nanodrugs,” ACS Nano 14(2), 1856–1865 (2020). ANCAC3 1936-0851 10.1021/acsnano.9b07993 31909985

[r14] Bianco V. et al., “Label-free intracellular multi-specificity in yeast cells by phase-contrast tomographic flow cytometry,” Small Methods 7(11), 2300447 (2023). 10.1002/smtd.202300447 37670547

[15] Kim M. et al., “Real-time monitoring of multitarget antimicrobial mechanisms of peptoids using label-free imaging with optical diffraction tomography,” Adv. Sci. 10(24), 2302483 (2023). 10.1002/advs.202302483 PMC10460844 37341246

[16] Saunders N. et al., “Dynamic label-free analysis of SARS-CoV-2 infection reveals virus-induced subcellular remodeling,” Nat. Commun. 15(1), 4996 (2024). NCAOBW 2041-1723 10.1038/s41467-024-49260-7 38862527 PMC11166935

[r17] Saba A. et al., “Physics-informed neural networks for diffraction tomography,” Adv. Photonics 4(6), 066001 (2022). AOPAC7 1943-8206 10.1117/1.AP.4.6.066001

[r18] Jo Y. et al., “Label-free multiplexed microtomography of endogenous subcellular dynamics using generalizable deep learning,” Nat. Cell Biol. 23(12), 1329–1337 (2021). NCBIFN 1465-7392 10.1038/s41556-021-00802-x 34876684

[r19] Ryu D. et al., “DeepRegularizer: rapid resolution enhancement of tomographic imaging using deep learning,” IEEE Trans. Med. Imaging 40(5), 1508–1518 (2021). ITMID4 0278-0062 10.1109/TMI.2021.3058373 33566760

[20] Choi G. et al., “Cycle-consistent deep learning approach to coherent noise reduction in optical diffraction tomography,” Opt. Express 27(4), 4927 (2019). OPEXFF 1094-4087 10.1364/OE.27.004927 30876102

[21] Wang K. et al., “On the use of deep learning for phase recovery,” Light Sci. Appl. 13(1), 4 (2024). 10.1038/s41377-023-01340-x 38161203 PMC10758000

[22] Kim G. et al., “Rapid species identification of pathogenic bacteria from a minute quantity exploiting three-dimensional quantitative phase imaging and artificial neural network,” Light Sci. Appl. 11(1), 190 (2022). 10.1038/s41377-022-00881-x 35739098 PMC9226356

[23] Ryu D. et al., “Label-free white blood cell classification using refractive index tomography and deep learning,” BME Front. 2021, 9893804 (2021). 10.34133/2021/9893804 37849908 PMC10521749

[24] Muhamad R. K. et al., “Off-axis image plane hologram compression in holographic tomography—metrological assessment,” Opt. Express 30(3), 4261–4273 (2022). OPEXFF 1094-4087 10.1364/OE.449932 35209666

[25] Guo Y. et al., “Deep learning for 3D point clouds: a survey,” IEEE Trans. Pattern Anal. Mach. Intell. 43(12), 4338–4364 (2021). ITPIDJ 0162-8828 10.1109/TPAMI.2020.3005434 32750799

[26] Phang J. T. S. Lim K. H. Chiong R. C. W., “A review of three dimensional reconstruction techniques,” Multimedia Tools Appl. 80(12), 17879–17891 (2021). 10.1007/s11042-021-10605-9

[r27] Fernandes D. et al., “Point-cloud based 3D object detection and classification methods for self-driving applications: a survey and taxonomy,” Inform. Fusion 68, 161–191 (2021). 10.1016/j.inffus.2020.11.002

[28] Wang Y. et al., “A point cloud-based deep learning strategy for protein–ligand binding affinity prediction,” Brief. Bioinform. 23(1), bbab474 (2022). 10.1093/bib/bbab474 34849569

[29] Zhang H. et al., “Deep learning-based 3D point cloud classification: a systematic survey and outlook,” Displays 79, 102456 (2023). DISPDP 0141-9382 10.1016/j.displa.2023.102456

[30] Lee M. Hugonnet H. Park Y., “Inverse problem solver for multiple light scattering using modified Born series,” Optica 9(2), 177 (2022). 10.1364/OPTICA.446511

[31] Lim J. et al., “Comparative study of iterative reconstruction algorithms for missing cone problems in optical diffraction tomography,” Opt. Express 23(13), 16933–16948 (2015). OPEXFF 1094-4087 10.1364/OE.23.016933 26191704

[32] Mazur M. Krauze W., “Volumetric segmentation of biological cells and subcellular structures for optical diffraction tomography images,” Biomed. Opt. Express 14(10), 5022 (2023). BOEICL 2156-7085 10.1364/BOE.498275 37854559 PMC10581803

[33] Cho M. J. et al., “Influence of chemical and genetic manipulations on cellular organelles quantified by label-free optical diffraction tomography,” Anal. Chem. 95(36), 13478–13487 (2023). ANCHAM 0003-2700 10.1021/acs.analchem.3c01349 37523497

[34] Kim H. et al., “Recent advances in label-free imaging and quantification techniques for the study of lipid droplets in cells,” Curr. Opin. Cell Biol. 87, 102342 (2024). COCBE3 0955-0674 10.1016/j.ceb.2024.102342 38428224

[35] Qi C. R. et al., “PointNet++: deep hierarchical feature learning on point sets in a metric space,” in Adv. in Neural Inform. Process. Syst. (NeurIPS), Curran Associates, Inc., pp. 5105–5114 (2017).

[36] Zhao H. et al., “Point transformer,” in IEEE/CVF Int. Conf. Comput. Vision (ICCV), pp. 16239–16248 (2021). 10.1109/ICCV48922.2021.01595

[37] Loshchilov I. Hutter F., “Decoupled weight decay regularization,” arXiv.1711.05101 (2017).

[38] Gotmare A. et al., “A closer look at deep learning heuristics: learning rate restarts, warmup and distillation,” arXiv.1810.13243 (2018).

[39] Zhu Q. Fan L. Weng N., “Advancements in point cloud data augmentation for deep learning: a survey,” Pattern Recognit. 153, 110532 (2024). 10.1016/j.patcog.2024.110532

[40] Jo M. et al., “Reduced dynamicity and increased high-order protein assemblies in dense fibrillar component of the nucleolus under cellular senescence,” Redox Biol. 75, 103279 (2024). 10.1016/j.redox.2024.103279 39111063 PMC11347067

[41] Huang G. et al., “Densely connected convolutional networks,” in IEEE Conf. Comput. Vision and Pattern Recognit. (CVPR), IEEE, pp. 2261–2269 (2017). 10.1109/CVPR.2017.243

[42] He K. et al., “Deep residual learning for image recognition,” in IEEE Conf. Comput. Vision and Pattern Recognit. (CVPR), IEEE, pp. 770–778 (2016). 10.1109/CVPR.2016.90

[43] Howard A. G. et al., “MobileNets: efficient convolutional neural networks for mobile vision applications,” arXiv.1704.04861 (2017).

[44] Sung M. et al., “Three-dimensional label-free morphology of CD8+ T cells as a sepsis biomarker,” Light Sci. Appl. 12(1), 265 (2023). 10.1038/s41377-023-01309-w 37932249 PMC10628166

[45] Lee M. J. et al., “Virtual biomarkers: predicting immune status using label-free holotomography of individual human monocytes and machine learning analysis,” bioRxiv 2023.09.12.557503 (2023).

