# Supplementary material for: Lightweight and precise cell classification based on holographic tomography-derived refractive index point cloud
Source: J Biomed Opt. 2025 Sep 2;30(9):096501. doi: 10.1117/1.JBO.30.9.096501 (PMC12404102; doi:10.1117/1.JBO.30.9.096501)
Supplement: Supplementary file 1 [file JBO_030_096501_SD001.pdf]

Supplementary information

Lightweight and precise cell classification based on holographic tomography-derived refractive index point cloud

Haoyuan Wang,[a,b,c] Difeng Wu,[a,b,c] Miao Zheng,[a,b,c] Zuoshuai Zhang,[a,b,c] Weina Zhang,[a,b,c] Jianglei Di,[a,b,c] and Liyun Zhong[a,b,c,*]

[a] Guangdong University of Technology, Institute of Advanced Photonics Technology, School of Information Engineering, Guangzhou, China

[b] Guangdong University of Technology, Key Laboratory of Photonic Technology for Integrated Sensing and Communication, Ministry of Education of China, Guangzhou, China

[c] Guangdong University of Technology, Guangdong Provincial Key Laboratory of Information Photonics Technology, Guangzhou, China

*Liyun Zhong**,** E-mail: zhongly@gdut.edu.cn

Fluorescence intensity distribution of cell viability.

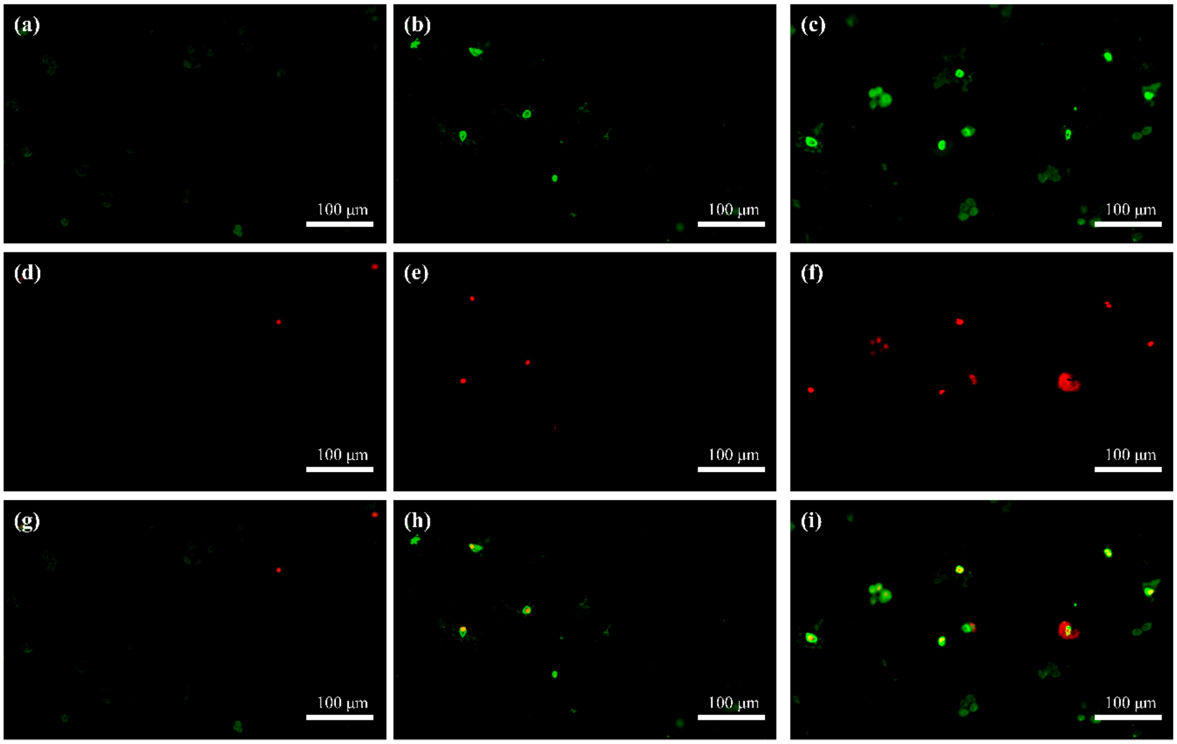

Fig. S1 Fluorescence intensity distribution of cell viability. Green fluorescence (a-c) denotes Annexin V－FITC staining, with the mean fluorescence intensity reflecting the degree of apoptosis;

red fluorescence(d-f) denotes propidium iodide (PI) staining, with the mean fluorescence intensity reflecting the degree of necrosis; merge image(g-i). (a) (d) (g) untreated control (high viability). (b) (e) (h) 3-hour treatment (moderate viability). (c) (f) (i) 6-hour treatment (low viability).

Classification performance metrics for varying point counts *K*

Table S1 Classification performance metrics for varying point counts *K*

Number of *K*	Accuracy	Precision	Recall	F1-score
512	0.848	0.849	0.856	0.852
1024	0.935	0.937	0.940	0.938
2048	0.935	0.934	0.941	0.937

Classification accuracy results for ten independent RICPS sampling tests

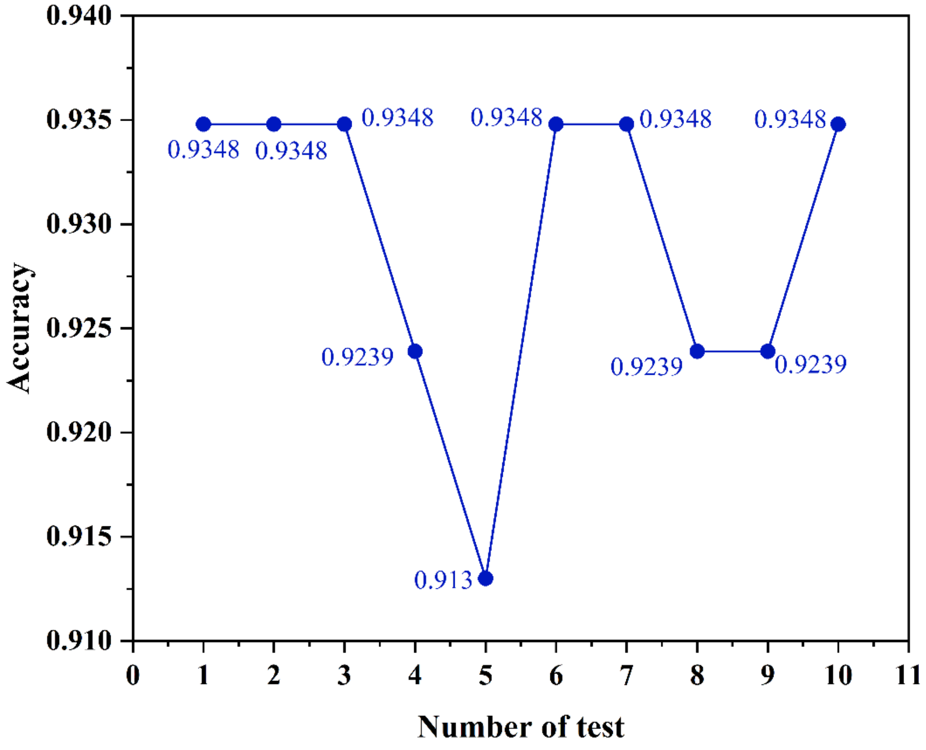

Fig. S2 Classification accuracy results for ten independent RICPS sampling tests.
